# In Situ Control of Reactive Mesogens Alignment During 3D Printing by Two‐Photon Lithography

**DOI:** 10.1002/advs.202415159

**Published:** 2025-03-31

**Authors:** Tiziana Ritacco, Alfredo Mazzulla, Michele Giocondo, Gabriella Cipparrone, Pasquale Pagliusi

**Affiliations:** ^1^ Department of Physics University of Calabria Rende (CS) 87036 Italy; ^2^ CNR Nanotec – Institute of Nanotechnology S.S. Cosenza Rende (CS) 87036 Italy; ^3^ CNR IMM – Institute for Microelectronics and Microsystems S.S. Roma Roma 00133 Italy

**Keywords:** 3D Printing, additive manufacturing, anti‐counterfeiting, liquid crystals, tunable alignment

## Abstract

Photopolymerizable liquid crystals, also known as reactive mesogens, are leading candidates for additive manufacturing of smart microdevices via two‐photon lithography (TPL). While substantial advancements are made toward innovative applications, precise control of molecular alignment during fabrication, essential for tailoring complex optical and mechanical responses, remains a significant challenge. Current solutions require elaborate multi‐step procedures or customized setups to achieve 2D or 3D alignment patterns. Herein, the deterministic effect of TPL on the orientation of mesogenic moieties is reported, under optimized printing conditions. Specifically, a single‐step simple method is developed for aligning the nematic director in situ, with sub‐diffraction‐limited resolution, during 3D printing. Based on the conventional TPL workflow, the “director‐tuning mode” (DiTuM) relies on the anisotropic photopolymerization reaction occurring along the print path at low laser scan speeds (≈0.1mm s^−1^). A TPL‐induced “easy axis” arises for the mesogenic moieties, programmable in direction and strength, and competes with the initial alignment to create potentially convolute 3D director fields. The method holds considerable promise for 3D/4D printing, enabling advanced functionalities, and offers a robust platform for anti‐counterfeiting applications, leveraging the unique optical signatures generated by complex microstructures.

## Graphical abstract

Manipulating material properties at the microscale is crucial for 3D printing of smart microdevices. Two‐photon lithography directly patterns molecular alignment in liquid crystal resists. The polymerization reaction wavefront along the writing path induces an easy‐axis for the mesogenic moieties. This competes with the rubbing‐induced alignment to create a programmable director and optical axis fields, enabling tailored functionalities.

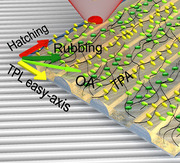

## 1 Introduction

Additive manufacturing of smart materials has undergone significant progresses in recent years, driven by its impactful applications in stimuli‐responsive systems, as well as in optics, photonics, and anti‐counterfeiting.^[^ ^1^, ^2^, ^3^, ^4^, ^5^, ^6^ ^]^ These advancements have been made possible by the integration of specific features or functionalities into 3D structures, via fabrication methods that precisely modulate the material's physical and/or chemical properties at the micro‐ or nanoscale.^[^ ^7^, ^8^, ^9^ ^]^

Liquid crystals (LCs),^[^ ^10^ ^]^ either as thermoplastic polymers/oligomers or photopolymerizable low‐molar‐mass mesogens (reactive mesogens), have emerged as a prominent class of functional materials for 3D printing of smart devices, due to the ability to self‐organize into different ordered phases, with tunable mechanical, electrical, magnetic, optical properties/anisotropies, and the sensitivity to external stimuli.^[^ ^1^, ^7^, ^8^, ^9^, ^10^, ^11^, ^12^, ^13^, ^14^, ^15^, ^16^, ^17^, ^18^ ^]^ Two categories of additive manufacturing techniques have been primarily employed with LCs, exhibiting both advantages and limitations. 3D extrusion‐based techniques, such as “fused filament fabrication” and “direct ink writing”, induce long‐range molecular order on polymer chains and mesogenic oligomers, due to the shear flow and temperature gradient along the print direction. However, they are limited to the fabrication of large‐scale objects because they lack the spatial resolution of optical lithographies.^[^ ^17^, ^18^ ^]^ Among the latter, two‐photon polymerization lithography (TPL) excels in high‐resolution fabrication of 3D complex structures, with submicrometric features (≈100 nm), in a variety of materials, including hydrogels, LCs, and organic/inorganic composites.^[^ ^12^, ^13^, ^16^, ^17^, ^18^, ^19^ ^]^

Harnessing the distinctive properties of reactive mesogens through TPL has yielded remarkable progress in the fields of soft microrobotics, microfluidics, microoptics, microphotonics, analytical sensing, and anti‐counterfeiting.^[^ ^1^, ^5^, ^11^, ^12^, ^13^, ^14^, ^15^, ^16^, ^17^, ^18^, ^20^, ^21^, ^22^, ^23^, ^24^, ^25^ ^]^ Nevertheless, realizing the full potential of additive manufacturing with LCs relies critically on achieving precise control over the molecular alignment (i.e., the director)^[^ ^10^ ^]^ in 3D, given its direct impact on the performance and functionality of the printed structures.^[^ ^8^, ^16^, ^17^, ^21^, ^22^ ^]^ Traditional methods for imposing uniform or nonuniform, albeit basic (i.e., twisted, splay), director fields involve surface‐mediated anchoring in alignment cells.^[^ ^10^ ^]^ A variety of modular approaches have emerged to implement arbitrary in‐plane 2D director fields, which include photoalignment lithography,^[^ ^22^ ^]^ micro‐ and nano‐rubbing techniques,^[^ ^26^ ^]^ microchannels created by direct laser writing.^[^ ^1^, ^8^, ^12^, ^13^, ^16^, ^17^, ^21^, ^27^ ^]^ However, surface‐based approaches lack the necessary point‐by‐point control over the director in 3D space, which limits the creation of complex architectures. Consequently, achieving more intricate 3D director arrangements requires multi‐step processes and/or customized setups, such as the assembly of printed components to combine different orientation domains,^[^ ^28^ ^]^ 3D microscaffolding,^[^ ^8^, ^29^ ^]^ or the application of electric fields during the printing process.^[^ ^30^, ^31^, ^32^ ^]^

In this context, the development of simple and functional methods for aligning the reactive mesogens director in 3D remains a key challenge. The ability to adjust the molecular alignment in situ and a single step would represent a significant breakthrough, as it would confer enhanced flexibility in patterning the director field, leading to advanced functionalities in the 3D/4D‐printed microstructures. However, unlike extrusion‐based techniques with LC elastomers,^[^ ^16^, ^17^ ^]^ a direct and deterministic influence of the TPL process on the mesogenic director orientation has not been demonstrated to date.

The alignment of reactive mesogens during scanning UV photopolymerization, namely SWaP, was recently reported by Shishido et al.^[^ ^33^ ^]^ The technique relies on spatiotemporal scanning of focused UV light to impose a local molecular order through diffusion‐induced mass flow relative to the scan direction.^[^ ^33^, ^34^, ^35^, ^36^, ^37^, ^38^ ^]^ However, the SWaP approach presents inherent shortcomings when it comes to the fabrication of 3D microdevices. In addition to the reduced spatial resolution (≈2 µm),^[^ ^33^ ^]^ which is limited by light diffraction, SWaP also lacks the 3D fabrication versatility offered by TPL, as it enables only 2D alignment patterns.

Here, we demonstrate that the director of nematic reactive mesogens (NRMs) can be controlled and spatially patterned in 3D by TPL, eliminating the need for external fields, surface patterns, or 3D scaffolds. The method employs the standard TPL workflow to reorient the NRMs director in situ during the printing process. Specifically, depending on parameters such as laser scan speed and direction, TPL induces an alignment “easy axis”, i.e., a preferred direction of molecular orientation, with tunable angle and strength. It competes with the initial NRMs director, both at the anchoring surface and in the bulk, enabling the creation of potentially convoluted 3D director and optical axis (OA) patterns.

In view of potential applications in authentication and anti‐counterfeiting, we explore the creation of optical signatures, which exhibit multiple levels of complexity and are hardly falsifiable. A 3D microtag is proposed as a security label, featuring an intricate polarized optical response. The latter is generated by the 3D director pattern, which is intentionally designed to have a complex correspondence with the microtag's small‐scale features, shape, and size.

## 2 Results and Discussion

**Figure 1:**
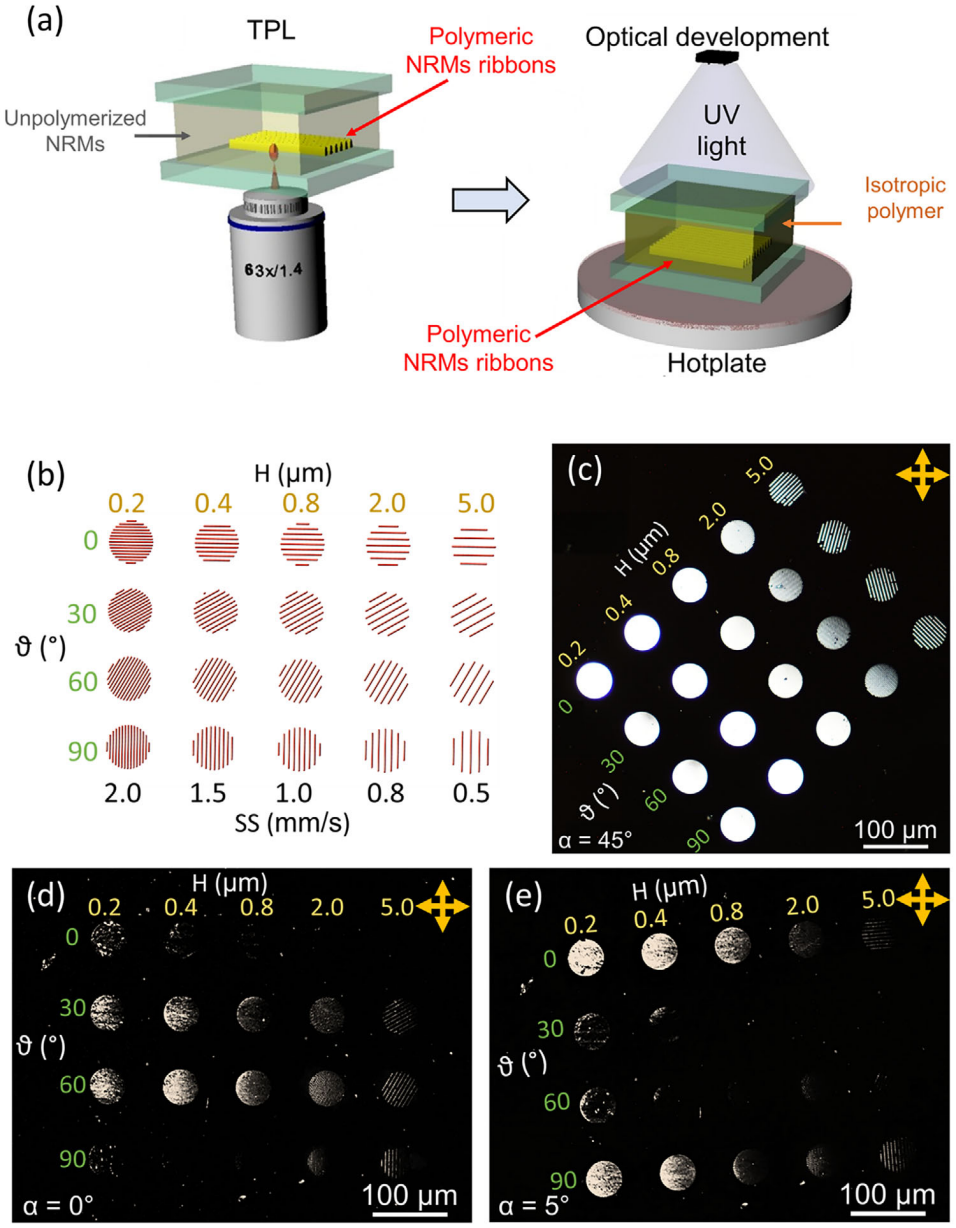
a) Schematic illustration of the TPL printing process within homogenously aligned NRMs cell (left) and of the subsequent “optical development” (right), where unreacted NRMs are UV‐cured above the clearing temperature, in the isotropic state. b) CAD of the single‐layer disks (diameter *d* = 50 µm) with different hatching distance H (top axis) and directions ϑ (left axis). The spacing between the hatching lines in the scheme is not to scale, but it has been expanded for the sake of clarity. All disks are printed at constant laser power LP = 17.5 mW. The scan speed, SS, (bottom axis) increases as H decreases (from 0.5 to 2.0 mm s^−1^) to ensure consistent fabricating conditions. c–e) Transmission optical micrographs of the TPL‐printed NRMs disks matrix embedded in the UV‐cured isotropic resist (i.e., optically developed), captured between crossed polarizers at 20× magnification. The micrographs illustrate the effect of varying the anchoring axis orientation with respect to the transmission axis of the input polarizer: (c) α = 45°, (d) α = 0° and (e) α = 5°.

The additive manufacturing process begins with a computer‐aided design (CAD) model of a 3D object, which is sliced into layers, and each layer hatched with parallel lines. When the latter are sequentially scanned by the focus of the ultrafast near‐infrared (NIR) laser in the photoresist, ellipsoidal polymer ribbons are generated by two‐photon absorption (TPA) photopolymerization. Their sub‐micrometric transverse dimensions (i.e., the voxel's height and width) depend on the exposure dose, which is, in turn, controlled by the average laser power LP and scan speed SS (see Figure S1 and Table S1, Supporting Information).^[^ ^19^, ^39^ ^]^ Consistent microstructures are fabricated by adjusting the slicing distance between layers and the hatching distance (H) between lines, according to the ribbons’ height and width, respectively.^[^ ^16^, ^17^, ^19^ ^]^ All the structures in this study are printed in uniform planar NRMs films, sandwiched between two alignment substrates with parallel anchoring axes (see **Figure** **1**a and Experimental Section).

In the following sections, we introduce a “director tuning mode” (DiTuM) for TPL, which leverages the printing parameters to locally reorient the director, while maintaining the nominal birefringence (see Figure S2, Supporting Information). First, DiTuM is investigated at the alignment substrate, by printing single‐layer structures in contact with it (Figure 1a). Afterward, multi‐layer and hanging single‐layer structures are reported to demonstrate the control of the director in the third, vertical, dimension.

An “optical development” protocol (see Figure 1a and Experimental Section), which exploits the thermotropic behavior of the NRMs, and polarized transmission microscopy are employed here to prove the DiTuM, unless otherwise stated.^[^ ^40^ ^]^ In contrast to conventional chemical development, the unpolymerized NRMs are not dissolved after TPL printing, but are UV‐cured in the isotropic state (i.e., above the nematic‐isotropic transition temperature). Consequently, the ribbon‐shaped TPL prints are embedded in the isotropic polymer film, which reduces the refractive index contrast and effectively prevents “form birefringence” (see Figure S3, Supporting Information).^[^ ^41^, ^42^, ^43^, ^44^, ^45^ ^]^ The orientational order of the mesogenic moieties remains the sole source of birefringence, thereby making the local OA indicative of the nematic director.

### 2.1 Reorienting the Director at the Alignment Surface

**Figure 2:**
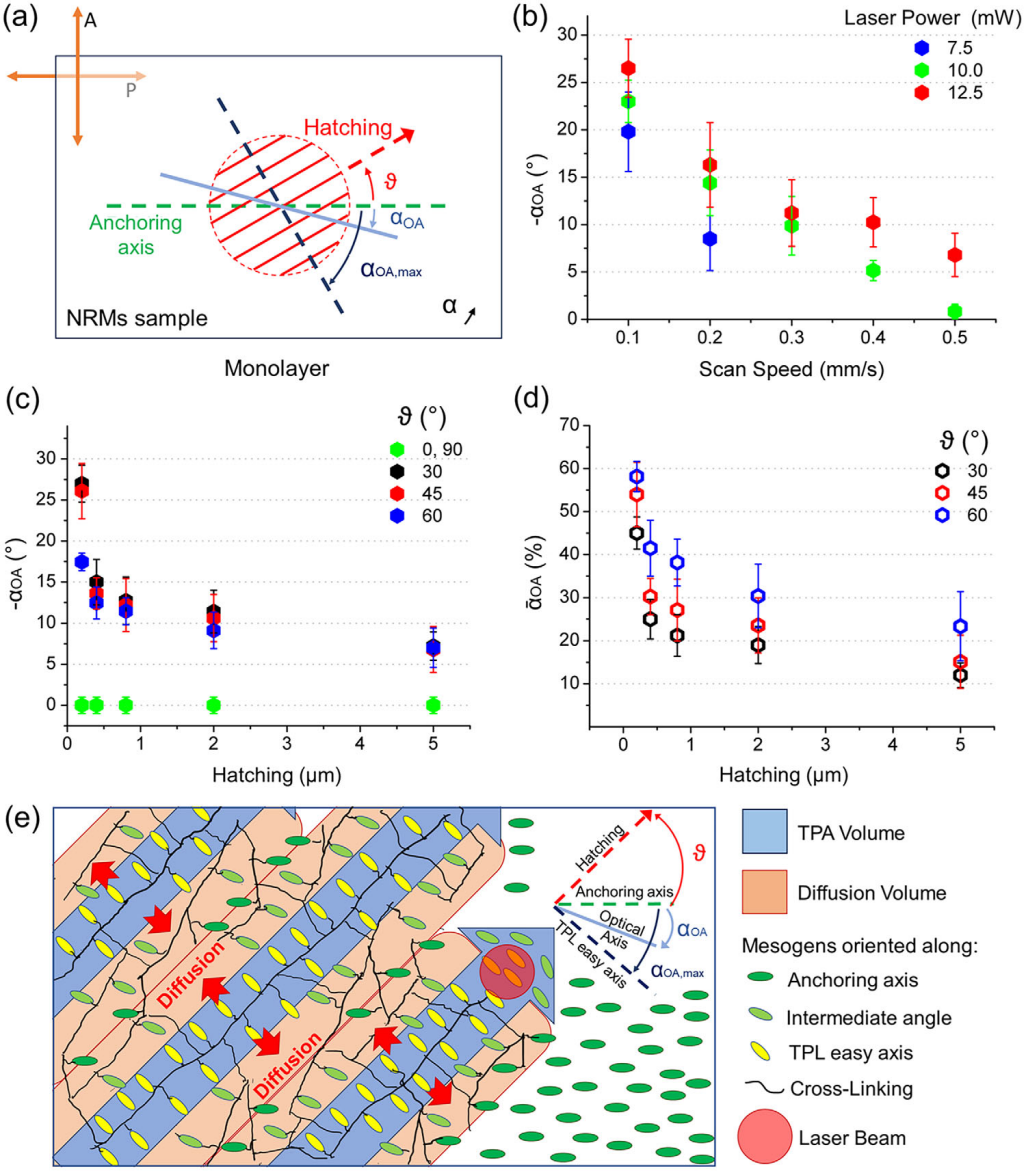
a) Schematic of the effective optical axis (cyan solid line) deviation α_*OA*_ which results from the competition between the TPL‐induced “easy axis” (blue dashed line), orthogonal to the hatching direction (red lines) ϑ and the anchoring axis (green dashed line). Clockwise (counterclockwise) angles measured from the anchoring axis are considered positive (negative). b) Influence of the scan speed (SS) and the laser power (LP) on the deviation of the OA/director α_*OA*_ for the NRMs disks hatched at ϑ = 45° and H = 0.2 µm. Higher OA/director deviations occur at lower SS and higher LP. c) Effect of the hatching distance H and direction ϑ on α_*OA*_ of the NRMs disks printed at SS = 0.1 mm s^−1^ and LP = 12.5 mW. Angular deviation decreases significantly as the hatching distance H increases above the ribbon width. d) Normalized OA/director angular deviation ${\tilde{\alpha }}_{OA}\equiv \alpha_{OA}/\alpha_{OA,max}$ increases with ϑ, for each H value, supporting the competition mechanism between the anchoring and the TPL‐induced “easy axes”. e) Schematic illustration of the TPL‐induced molecular reorientation (TPL DiTuM). When the NIR ultrafast laser focus (red circle) is scanned at low SS values two‐photon absorption process occurs in narrow ribbons (TPA volume), where free radicals are generated, and a chain reaction is initiated. Molecular flow aligns the polymer main‐chains along the hatching direction, while mesogenic moieties (in yellow) aim at perpendicular orientation (α_*OA*, *max*_). Polymerization also extends into the adjacent “diffusion volume” where mesogenic moieties adopt intermediate angles (light green) and relaxes toward the anchoring axis (dark green) with increasing distance from the TPA volume.

As a first step, a test matrix consisting of single‐layer microdisks (diameter *d* = 50 µm) is designed by varying the hatching distances H and angles ϑ with respect to the anchoring axis imposed by the aligning surface (Figure 1b). To ensure consistent fabrication conditions, the disks with smaller hatching distances are scanned at higher speeds (see lower horizontal scale in Figure 1b). This compensates for the cumulative heat load and thermal polymerization associated with denser infill patterns, preventing overexposed ribbons, bubbling, and burning. The disks, printed at the NRMs‐substrate interface and “optically developed”, are analyzed by cross‐polarized transmission microscopy, for different orientations α of the anchoring axis with respect to the polarizers (Figure 1c–e). The brightness of the disks at α = 45° against the dark background of the isotropic polymer (Figure 1c) proves their optical anisotropy, with comparable birefringence values across the explored range of TPL parameters (see Figure S2, Supporting Information).^[^ ^9^, ^46^ ^]^ However, only the disks hatched parallel (ϑ = 0°) and perpendicular (ϑ = 90°) to the anchoring axis maintain the OA imposed by the alignment substrate, as evidenced by their minimum transmission at α  =  0° (Figure 1d). The disks hatched at intermediate angles (ϑ = 30 and 60°) are noticeably brighter at α  =  0°. They manifest the extinction condition at α≅5° (Figure 1e) suggesting that their OA has undergone a clockwise (negative) rotation of α_*OA*_ =   − α≅ − 5°, away from the hatching direction (**Figure** **2**a).

Further analysis of TPL parameters confirms the initial observation of such slight in‐plane director reorientation. First, the impact of SS is evaluated by printing solid (H = 0.2 µm) single‐layer disks with a constant hatching direction ϑ = 45°. Significant director deviations are achieved as SS decreases, reaching α_*OA*_≅ − 26° for SS = 0.1 mm s^−1^ (LP = 12.5 mW) (Figure 2b). Second, the influence of the hatching orientation ϑ and distance H on α_*OA*_ is investigated, while maintaining the aforesaid SS and LP values constant (Figure 2c,d). While it is confirmed that the disks printed at ϑ = 0° and 90° preserve the alignment of the uncured NRMs film, the OA deviation for intermediate ϑ values rapidly decreases with the hatching distance H (Figure 2c).

In view of the reported results, we propose that a TPL‐induced “easy axis” for the mesogenic moieties arises when printing is performed at low laser scan speeds. It is perpendicular to the hatching direction (i.e., α_*OA*, *max*_ =  ϑ − 90°) and competes with the surface‐mediated anchoring in defining the local director. To assess the efficacy of the TPL‐induced “easy axis”, it is advantageous to consider the normalized deviation angle of the OA/director, defined as ${\tilde{\alpha }}_{OA}\equiv \alpha_{OA}/\alpha_{OA,max}$. Its values, based on data in Figure 2c, increase with the hatching angle ϑ in the [30°, 60°] interval, for any hatching distance (Figure 2d). For the denser infill pattern (H = 0.2 µm), ${\tilde{\alpha }}_{OA}$ ranges between 45% and 60%. The most efficient OA/director reorientation occurs for the structures hatched at ϑ = 60°, due to the lower TPL‐induced “easy axis” angle α_*OA*, *max*_ (60°) =   − 30°.

Figure 2e provides a schematic representation of the potential molecular‐scale mechanisms underlying the TPL‐induced director reorientation. This illustration emphasizes the preferential alignment of mesogenic moieties with respect to the scanning direction ϑ and the influence of the hatching distance H. As the NIR laser focus (red circle) moves through the NRMs, free radicals are generated in an ellipsoidal TPA volume, with sub‐diffraction‐limited transverse dimensions, and a chain photopolymerization reaction is initiated. In the low SS regime, it also establishes a chemical potential gradient along the hatching direction, similar to scanning UV light in the SWaP process.^[^ ^33^, ^34^, ^35^, ^36^, ^37^, ^38^ ^]^ The resulting mass flow, driven by molecular diffusion, induces a shear stress that aligns the polymer main‐chains along the scanning direction (i.e., parallel to the ribbons). The preferential orientation of mesogenic moieties relative to the polymer main‐chain is dictated by their molecular structure, especially the length of the alkyl spacers. Specifically, recent literature indicates that long alkyl spacers promote parallel alignment, whereas short spacers favor perpendicular alignment.^[^ ^36^ ^]^ In this study, the observed TPL “easy axis” indicates that the mesogenic side‐chain units (yellow in the scheme) within the TPA volume exhibit a preferential alignment orthogonal to the polymer main‐chains.

The effect of the hatching spacing H on the average OA/director reorientation, as depicted in Figure 2c,d, is attributed to polymerization propagating beyond the TPA volume, via free radical diffusion.^[^ ^47^, ^48^, ^49^, ^50^ ^]^ A reduced director deviation (light green, in Figure 2e) occurs in the outer shell of the polymeric ribbon (Diffusion volume) as a result of competition with surface anchoring (dark green).^[^ ^10^, ^51^ ^]^ Due to its inherent spatial resolution limitation, polarized optical microscopy yields a spatially averaged OA/director orientation across the entire photopolymerized ribbon (width ≈0.30 µm and height ≈0.20 µm, see Figure S1 and Table S1, Supporting Information), integrating contributions from both the TPA and diffusion regions. As the hatching spacing is reduced, approaching the TPA volume width, the diffusion volume is constrained, leading to a corresponding increase in the average OA/director deviation (Figure 2c). Nonetheless, even at the lowest investigated hatching spacing (H = 0.2 µm), the reorientation remains below the predicted maximum α_*OA*, *max*_, i.e., ${\tilde{\alpha }}_{OA}\leq 60\%$ (Figure 2d). This finding suggests that the anchoring conditions restrict the director's capability to fully line up with the TPL “easy axis” in single‐layer structures printed on the alignment substrate.

### 2.2 Twisting the Director in Multi‐Layer Structures

**Figure 3:**
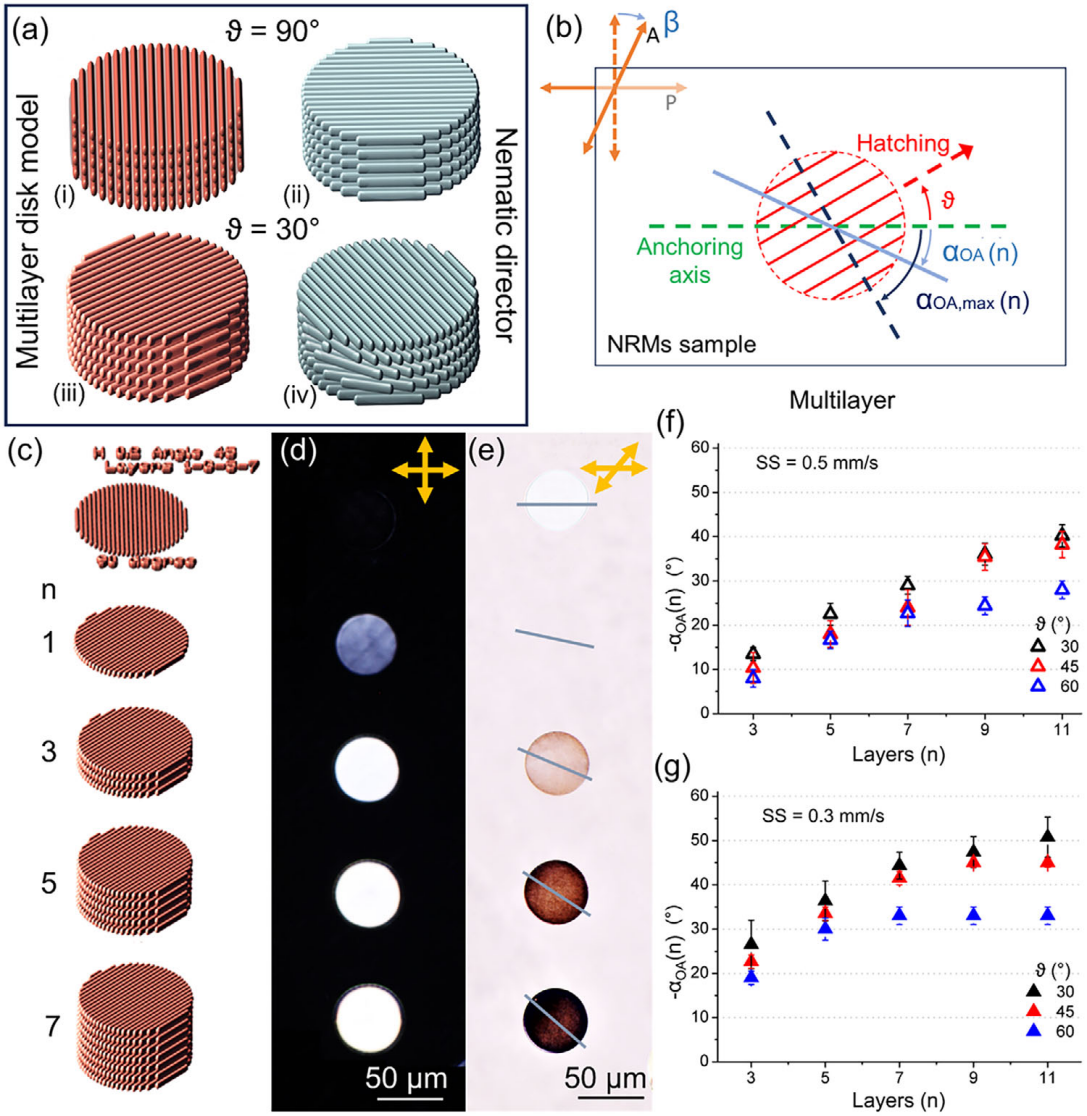
a) CAD model of multi‐layer microcylinders hatched at ϑ = 90°, 30° (i, iii) and schematic illustration of the corresponding uniform (ii) and twisted (iv) nematic director fields. The microcylinder hatched at ϑ = 90° (i) maintains the homogeneous director, parallel to the anchoring axis (ii). Conversely, at ϑ = 30° (iii) microcylinders exhibit a twisted nematic configuration along the *z*‐axis (iv). b) Schematic illustration of the optical axis (cyan solid line) deviation α_*OA*_ (*n*) of the topmost *n*‐th layer. The analyzer is rotated by β  = α_*OA*_ (*n*) in order to minimize the transmitted linearly polarized light guided through the twisted nematic structure. c) CAD model of the microcylinders (d = 50 µm, H = 0.2 µm, S = 0.5 µm, ϑ = 45°) with *n* ≤ 7 layers. A reference single‐layer disk hatched at ϑ = 90° is located on the first row. Note that the spacing between hatching lines in the CAD models is not in scale, but it has been expanded for clarity. d,e) Transmission polarized optical micrographs at 20× magnification of the multi‐layer cylinders array printed in the NRMs film (LP = 12.5 mW and SS = 0.3 mm s^−1^), and optically developed. The array is observed between crossed polarizers (d) and with the analyzer rotated by ‐40° (e). The cyan lines in Figure 4e denote the OA/director of the topmost layer of each structure. f,g) The OA/director rotation − α_*OA*_ (*n*) of the top layer is plotted versus the number of layers *n*, for ϑ = 30, 45, and 60°, and SS = 0.5 mm s^−1^ (f) and 0.3 mm s^−1^ (g).

To alleviate the constraints imposed by surface anchoring,^[^ ^10^, ^51^ ^]^ and enhance the OA/director deviation toward the TPL‐induced “easy axis”, a viable strategy involves printing at a distance from the substrate. As a next step, the TPL DiTuM is exploited to print 3D microstructures with a vertically varying director field, achieved by stacking parallel layers from the substrate. For simplicity, we consider solid cylinders composed of identical disk‐shaped layers, as depicted in Figures 2a and **3**a. Within each cylinder, all layers exhibit the same hatching pattern (H and ϑ) and laser printing (LP and SS) parameters, such that each structure is homogeneous “by design” along its height (i.e., *z*‐axis). On the other hand, the local OA/director of the *n*‐th layer is expected to progressively align with the TPL‐induced “easy axis” as *n* increases, moving further from the substrate. Figure 3a provides a schematic 3D illustration of multi‐layer structures hatched at ϑ = 90° and 30° (red lines), along with their corresponding OA/director fields (cyan lines). When hatching lines are perpendicular to the anchoring axis, i.e., ϑ = 90° (i), each layer maintains the alignment of the uncured NRMs film, resulting in a homogeneous OA/director field (ii). Conversely, at ϑ = 30° (iii) the OA/director rotates layer‐by‐layer (iv), forming a twisted nematic configuration^[^ ^10^, ^52^ ^]^ and gradually approaching the maximum rotation value α_*OA*, *max*_ as the number of layers increases.

The OA/director rotation α_*OA*_ (*n*) of the topmost *n*‐th layer can be estimated through polarized optical microscopy (Figure 3b). In slow‐twist regime (i.e., *d*α_*OA*_/*dz* ≪ 2π/λ, where λ is the light wavelength), also known as the Mauguin regime, the director fields guides the light polarization.^[^ ^52^ ^]^ Therefore, with the anchoring axis aligned parallel to the entrance polarizer axis (P), minimum light transmission occurs when the analyzer (A) is rotated by β  = α_*OA*_ (*n*) from the crossed configuration (Figure 3b), as it becomes orthogonal to the director of the topmost layer.

The director deviation α_*OA*_(*n*) in the *n*‐th layer is measured as a function of the number *n* of layers, for three hatching directions (ϑ = 30, 45, 60°), and two laser scan speeds (SS = 0.3 and 0.5 mm s^−1^), while the other parameters are kept constant (H = 0.2 µm, S = 0.5 µm, LP = 12.5 mW). Figure 3c illustrates the CAD of a microcylinder array with ϑ = 45°. A single‐layer disk hatched at ϑ = 90° is printed atop each array to serve as reference. Figure 3d,e shows the polarized optical micrographs of the microcylinders’ array for different orientation of the analyzer A. In Figure 3d, where the sample is imaged between crossed polarizers (β = 0°), all the structures, except for the reference disk, are bright because of the twisted director configuration. A rotation of the analyzer by β≅ − 40° ensures the extinction condition for the bottom cylinder (*n* = 7) (see Figure 3e). Notably, the director approaches the TPL‐induced “easy axis” angle α_*OA*, *max*_ (45°) =   − 45° within only seven layers, achieving a normalized deviation ${\tilde{\alpha }}_{OA}≅89\%$.

Figure 3f,g proves that the overall director twist of the microcylinders increases with the number of layers *n*. For SS = 0.5 mm s^−1^, only the director of the structures hatched at ϑ = 60° aligns with the TPL‐induced “easy axis” angle α_*OA*, *max*_ (60°) =   − 30°, for *n* = 11. Far from the saturation regime, i.e., for *n* ≤ 7, the director twist rate *d*α_*OA*_/*dn* ∝ *d*α_*OA*_/*dz* exhibits minimal dependence on the hatching angle ϑ (Figure 3f). For the lower scan speed SS = 0.3 mm s^−1^, the twist rate increases (Figure 3g). Microcylinders hatched at ϑ = 60° and 45° exhibit saturation for the topmost layer's director already for *n* > 5 and *n* > 7, respectively, while the structures hatched at ϑ = 30° would require more than 11 layers for the director to line up with the TPL‐induced “easy axis”.

### 2.3 Precise Control of the Director by 3D Printing in the Bulk

**Figure 4:**
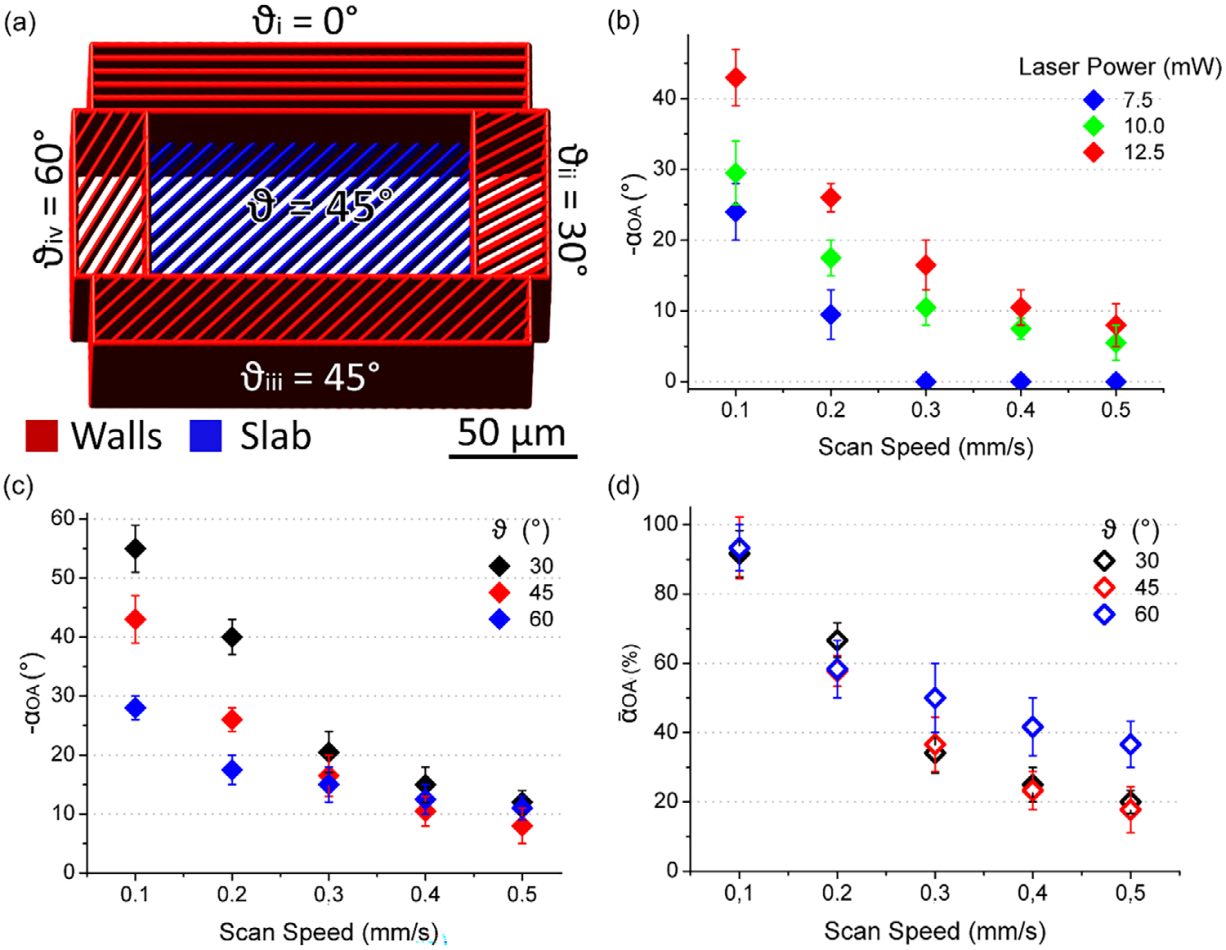
a) Schematic CAD model of the 3D *walls‐slab* test structure. The 6 µm tall *walls* (in red) are sliced and hatched at S = 0.35 µm, H = 0.4 µm and ϑ_i‐iv_ = 0, 30, 45 and 60°. They are printed at SS = 4.0 mm s^−1^ and LP = 15 mW. The single‐layer *slab* (in blue), located at the mid‐height of the *walls*, is hatched at H = 0.2 µm and ϑ = 30, 45 or 60° (ϑ = 45° is depicted in the scheme). Note that the spacing between hatching lines in the illustration is not to scale, but is expanded for clarity. b) Influence of the laser scan speed (SS) and power (LP) on the OA/director deviation − α_*OA*_ of the *slabs* hatched at ϑ = 45°, as evaluated by cross‐polarized optical microscopy. c) The OA/director rotation − α_*OA*_ of *slabs* hatched at ϑ = 30, 45, and 60° increases as the SS is decreased (LP = 12.5 mW is kept constant). Its highest values 55° ± 4°, 43° ±4 ° and 28°±2°, achieved at SS = 0.1 mm s^−1^, nearly match the corresponding TPL “easy axis” angles − α_*OA*, *max*_ (ϑ) =  90° − ϑ, for ϑ = 30, 45, and 60°, respectively. d) The normalized OA/director rotation ${\tilde{\alpha }}_{OA}$ varies from 19% to 94% across the investigated printing parameters space. At the lowest SS value, the director closely aligns with the TPL‐induced “easy axis” for all investigated hatching angles ϑ.

Here we demonstrate a high degree of control over the director in 3D, ultimately achieving the complete alignment with the TPL‐induced “easy axis”. This is accomplished by printing a sub‐micron‐thick layer directly in the NRMs bulk, effectively eliminating surface anchoring constraints. A 3D test structure, consisting of four supporting *walls* and a suspended single‐layer *slab*, has been designed for this purpose (**Figure** **4**a). By comparing the suspended *slabs* (Figure 4b) with the disks printed in contact with the alignment substrate (Figure 2b), both hatched at H = 0.2 µm and ϑ = 45°, one can directly evaluate the effectiveness of the bulk printing in controlling the director. Complete director reorientation along the TPL‐induced “easy axis” (i.e., − α_*OA*_≅45°) is attained for the *slab* printed at SS = 0.1 mm s^−1^ and LP = 12.5 mW, whereas the corresponding substrate‐bound disk only reaches 26°. Similarly, the OA/director in *slabs* hatched at 30° and 60° nearly matches the corresponding maximum angles α_*OA*, *max*_ (Figure 4c). Normalized deviations ${\tilde{\alpha }}_{OA}$ consistently exceed 90% at SS = 0.1 mm s^−1^ for all the three hatching angles and often reach 100% within the experimental error (Figure 4d).

Notably, in addition to the pursuit of optimal alignment with the TPL “easy axis”, these findings demonstrate that the director, whether in the substrate‐bound (Figure 2b) or suspended single‐layer (Figure 4b,c), or in the twisted configuration of multi‐layered structures (Figure 3f,g), exhibits extensive angular tunability achieved by the SS and LP parameters.

### 2.4 Security Microtag

The ability to control the director, regardless of the 3D print shape, size, and structural features such as infill patterns, offers significant advantages for advanced manufacturing, including 4D printing, and applications in optics and photonics. Here, we discuss the benefits of the TPL DiTuM for authentication and anti‐counterfeiting. The potential for encoding the OA of individual TPL‐polymerized NRMs ribbons, irrespective of the hatching direction, represents a key feature that enables the development of smart optical microlabels. As demonstrated in preceding sections, local OA/director orientation is strongly influenced by laser scan speed and power (SS and LP), manufacturer‐specific printing parameters that, in contrast to the hatching ones (H and ϑ), are typically undisclosed and not readily available. Consequently, the TPL DiTuM introduces an additional layer of optical information to the structure, easily detectable in polarized light, thus paving the way for 3D smart microtags with multi‐level encryption.

**Figure 5:**
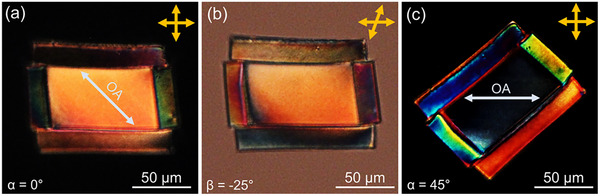
Transmission polarized optical micrographs at 50× magnification of the 3D *wall‐slab* structure, whose CAD is sketched in Figure 4a, printed in NRMs, and subsequently optically developed. a) The sample is oriented with the anchoring axis parallel to the input linear polarization P (α  =  0°) and imaged between crossed polarizers (A ⊥ P) or b) with the analyzer A rotated by β  =   − 25°. c) When the sample is rotated by α  =  45° and imaged between crossed polarizers (A ⊥ P), the *slab* appears dark, while the *walls* demonstrate a wide color gamut, contingent on their hatching angles and the TPL printing parameters employed.

For example, the 3D *walls*‐*slab* structure illustrated in Figure 4a already represents a convoluted security microtag with encoded optical information. Its complexity arises from the independent twisted nematic arrangements of the *walls*, which are hatched at distinct angles ϑ_i‐iv_. **Figure** **5** shows the transmission polarized optical micrographs of the structure, captured at varying sample and analyzer orientations. The *slab*, hatched at ϑ = 45°, exhibits a fully reoriented OA/director (α_*OA*_ =   − 45°), due to the printing parameters SS = 0.1 mm s^−1^ and LP = 12.5 mW. This is evidenced by the brightest and darkest conditions observed between crossed polarizers at α  =  0° and 45°, respectively (Figure 5a,c). On the other hand, the *walls* exhibit the polarization‐guiding effect characteristic of twisted nematic structures, consistent with the microcylinders shown in Figure 3. First, rotating the analyzer modulates the light transmission, as shown in Figure 5a,b, where the bottom *wall*, hatched at ϑ_iii_ = 45°, changes from bright to dark when the analyzer is rotated by 25°. Second, rotating the sample between crossed polarizers (see Figure 5c), reveals a vibrant color code due to the different twists of the *walls* and the material's birefringence dispersion.

It is worth noting that, even though the *walls* consist of several layers (*n* = 18), the director of the topmost layer does not completely align with the TPL‐induced “easy axis” (e.g., |β| < 45° for ϑ_iii_ = 45°, in Figure 5b), because of the high laser scan speed employed (SS = 4.0 mm s^−1^). Precise control over the *walls’* twist rate and *slab*’s OA/director, through SS and LP, introduces additional layers of complexity and security to the structure. By decoupling the optical response from its finest spatial features, i.e., the direction of the polymer ribbons, the *walls‐slab* microtag becomes unique and virtually impossible to reproduce, even if the underlying geometric parameters (H, S, ϑ) of the CAD were disclosed.

## 3 Conclusion

This study introduces a method for controlling the alignment of reactive mesogens, during additive manufacturing with two‐photon photopolymerization lithography (TPL). The “director‐tuning mode” (DiTuM) relies on the TPL printing parameters to achieve direct, single‐step patterning of the director in all three spatial dimensions, thereby circumventing the necessity for external driving fields, intricate surface alignment, 3D microscaffolds or other multi‐step fabrication procedures.

We have presented a thorough polarized optical microscopy investigation to assess the influence of the fabrication parameters on the alignment of the mesogenic moieties during TPL in planar NRMs cells. Specifically, the capability of the TPL DiTuM to reorient the director has been demonstrated in single‐ and multi‐layer structures, printed in contact with the anchoring surface, as well as in overhanging *slabs*, printed within the NRMs bulk. In the low laser scan speed regime (SS ≈ 0.1 mm s^−1^), TPL has been proved to induce an “easy axis” for the alignment of mesogenic moieties. The orientation of this axis is dictated by the laser scan direction, while its strength can be adjusted by controlling the laser power and scan speed. The director field in the printed microstructures results from the interplay between the TPL‐induced “easy axis” and the initial molecular alignment in the uncured NRMs film. Surface anchoring limits director reorientation in submicron‐thick (≈0.20 µm) single‐layers adjacent to the alignment substrates, resulting in normalized OA/director deviation ${\tilde{\alpha }}_{OA}\leq 60\%$. However, larger molecular deviation can be achieved when the microstructures are printed in the bulk, away from the aligning substrates, where the director can nearly align with the TPL “easy axis” (${\tilde{\alpha }}_{OA}\leq 94\%$) even in a single‐layer *slab*. By leveraging this competitive effect, we have successfully demonstrated uniformly hatched multi‐layer structures that exhibit twisted director arrangements along the vertical direction, with tunable twist rates and a fully reorientable topmost layer.

The proposed mechanism behind the TPL DiTuM is founded upon the anisotropic crosslinking reaction, mediated by a gradient of photoactivated species established along the laser scanning direction. Low values of the laser scan speed promote anisotropic molecular diffusion and directional photopolymerization, which ultimately lead to the reorientation of mesogenic moieties. TPL DiTuM shares conceptual similarities with the SWaP method, proposed by Shishido et al.,^[^ ^33^, ^34^, ^35^, ^36^, ^37^, ^38^ ^]^ in term of the physico‐chemical phenomena behind molecular alignment. Nevertheless, it offers a significant advantage by enabling 3D patterning of molecular alignment during 3D printing with sub‐diffraction‐limited spatial resolution (typical ribbon width ≈0.30 µm and height ≈0.20 µm), whereas SWaP is limited to 2D director patterning with minimum feature width one order of magnitude larger (≈2 µm).

It is important to note that the “optical development” process, which embeds the TPL‐fabricated birefringent microstructures within the same resist UV‐cured in the isotropic state, has been implemented here solely to simplify the analysis of director reorientation using polarized optical microscopy. This approach avoids the contribution of geometric optical anisotropy (form birefringence),^[^ ^41^, ^42^, ^43^, ^44^, ^45^, ^53^ ^]^ which could affect chemically developed TPL prints due to the filamentary structure of each layer, and it ensures that the orientation of the mesogenic moieties is the sole cause of the observed birefringence and optical axis. However, the proposed molecular‐scale mechanism suggests that the director‐tuning capability of TPL is independent of the development method.

To proof test the potential of the technique in the fields of authentication and anti‐counterfeiting, an optical microtag is reported here. Due to its complex 3D director architecture, the angular‐dependent response in polarized optical microscopy intricately correlates with its structural features, such as slicing distance, infill density, and direction. Therefore, it represents a hard‐to‐replicate optical security code with enhanced multi‐level encryption.

## 4 Experimental Section

### Materials and Reagents

Propylene glycol monomethyl ether acetate (PGMEA, ≥99.5%), isopropanol (≥99.5%), acetone (≥99.5%), and polyvinyl alcohol (PVA, 98% hydrolyzed, molecular weight: 13000–23000 g mol^−1^) were purchased by Sigma Aldrich. The photoinitiator phenylbis(2,4,6‐trimethylbenzoyl)‐phosphine oxide (Irgacure 2100) was supplied by Ciba Specialty.

### Photoresists

The liquid crystalline photoresist (NRMs) was prepared from a commercial solution (Licrivue RMs03‐001C, Merck) of four mono‐ and di‐acrylates mesogens dissolved, 30% by weight, in PGMEA.^[^ ^46^ ^]^ The reactive mesogens in the mixture are: 4‐(6‐ acryloyloxyhexyloxy)‐benzoic acid (4‐cyanophenyl ester) (RM 28), 2‐methyl‐1,4‐ phenylene‐bis[4‐(6‐acyloyloxyhexyloxy)benzoate] (RM 82), 4‐(3‐loxy)‐benzoic acid 2‐methyl‐1,4‐phenylene ester (RM 257) and 4‐(6‐acryloyloxyhexyloxy)‐benzoic acid‐ (4‐methoxyphenylester) (RM 105).^[^ ^9^ ^]^ The RMs03‐001C solution was left for 24 h at 80 °C in a vacuum chamber (0.3 × 10^−3^ mbar) to allow for complete evaporation of the solvent. The nematic‐isotropic transition of the dry unpolymerized NRMs occurs at 75 °C. Upon UV irradiation or TPL, the photoinitiators decompose into free radicals, starting a chain reaction of the reactive acrylate tails, which results in the creation of a cross‐linked network of carbon‐carbon bonds. The NRMs exhibit ordinary and extraordinary refractive indices n_o_ = 1.529 ± 0.005 and n_e_ = 1.684 ± 0.005, as measured at wavelength 589 nm and temperature 20 °C, after UV photopolymerization (wavelength 365 nm, intensity 20 mW cm^−2^, irradiation time 60 s).^[^ ^46^ ^]^ The photoinitiator phenylbis(2,4,6‐trimethylbenzoyl)‐phosphine oxide (Irgacure 2100) was added to the dry NRMs (3 wt.%) to increase TPA cross‐section and promote the free radical polymerization in TPL.

### Substrates and Cells

The NRMs mixture was confined within 10µm‐thick pi‐cells to obtain uniaxial planar alignment.^[^ ^10^, ^54^ ^]^ Each cell was assembled with a microscope slide (15 × 15 × 1.1 mm^3^) and a coverslip (22 × 22 × 0.17 mm^3^). Both microscope slides and coverslips underwent a cleaning procedure involving a 5 min ultrasonic bath in acetone, followed by a rinse with isopropanol. A thin PVA film, ≈20 nm thick, was layered on both the microscope slides and the coverslips. This was achieved by spin coating a PVA aqueous solution (1 wt.%, filtered at 0.2 µm and stirred for 2 h) at 3600 rpm for 1 min and baking the films for 40 min at 105 °C to allow for water evaporation. After cooling down to room temperature, the PVA films were bidirectionally rubbed by a velvet roll to promote planar anchoring of the nematic RMs.^[^ ^54^ ^]^ The coated substrates were assembled with the PVA films facing each other and with parallel rubbing directions. Two 10‐µm‐thick Mylar spacers were placed to create a uniform gap between the substrates, which were glued together at the corners with a small amount of UV‐curing adhesive (NOA 65, Norland).

The dry NRMs mixture, previously stirred for 1 h at 90 °C, was infiltrated by capillarity into the cell in the isotropic state. Then, it was slowly cooled down in the nematic phase at room temperature.

### Computer Design of the Structures

All the structures were designed using the commercial computer‐aided design (CAD) software Rhinoceros 5.0. The CAD files were converted to the STL file format and imported into the computer‐aided manufacturing software DeScribe 19.0 to generate the codes for the laser printing path. The multi‐layered structures were sliced in horizontal planes, i.e., parallel to the cell substrates, every 0.35 or 0.5 µm, according to the average height of the voxel/ribbons. The same infill pattern was adopted for both single‐ and multi‐layer structures, made of parallel lines at different hatching distances H ∈ [0.1, 5.0] µm and angles ϑ ∈ [0°, 90°] with respect to the anchoring axis. Consecutive planes of the same multi‐layer structure shared the same hatching parameters H and ϑ.

### Two‐Photon Lithography

TPL was performed using a commercially available workstation (Photonic Professional GT, Nanoscribe GmbH) equipped with an erbium‐doped fiber femtosecond laser oscillator/amplifier, followed by a second harmonic generation module (central wavelength: 780 nm, pulse duration: ≤100 fs, repetition rate: 80 MHz, max average power: 140 mW). The laser beam was attenuated by an acousto‐optic modulator to the desired average back focal plane power (LP ∈ [7.5, 17.5] mW), circularly polarized, and coupled to an inverted microscope (Axio Observer Z1, Zeiss), through a galvo‐mirror scanner. The laser beam was focused with a 63 × oil‐immersion objective (Plan‐Apochromat 63 ×/1.4 oil DIC M27, Zeiss) through the coverslip into the filled cell. A piezo translation stage was used to set the sample position with respect to the focal plane (i.e., along the vertical *z*‐axis) and control the slicing distance in the multi‐layer structures. The galvo stage was employed to sweep the laser focus along the hatching lines in the xy‐plane, with scan speed (SS) in the range [0.1, 4.0] mm s^−1^. To ensure optimal adhesion of both single‐ and multi‐layer structures with the coverslip, fabrication was initiated at coverslip/photoresist interface (as automatically detected by the Zeiss Definite Focus system), so that the ellipsoidal focal volume is halfway in the substrate.

### Sample Development

Following the TPL session, an “optical development” process was employed in lieu of dissolving the unexposed resist by wet chemical etching, unless otherwise specified. The NRMs cells were placed on a hotplate at an initial temperature of 60 °C and heated gradually to 80 °C (heating rate 0.2 °C s^−1^), which is above the NMRs clearing temperature. Subsequently, the cells were then uniformly exposed to a UV source (peak emission wavelength 365 nm, for 60 s at 24 mW cm^−2^) to promote homogeneous polymerization of the uncured NRMs in the isotropic phase.

### Optical Microscopy and Analysis

Optical micrographs were recorded in transmission mode on a polarizing upright microscope (Axio Scope A1, Zeiss) equipped with a halogen lamp (HAL 100), 20×/NA 0.50 or a 50×/NA 0.70 objective (Epiplan POL, Zeiss) and a DSLR camera (EOS 600D, Canon). All micrographs were collected in polarized light, either between crossed linear polarizers (A ⊥ P) or by rotating the analyzer A.

## Conflict of Interest

The authors declare no conflict of interest.

## Supporting information



Supporting Information

## Data Availability

The data that support the findings of this study are available from the corresponding author upon reasonable request.
