# Supplementary material for: In Situ Control of Reactive Mesogens Alignment During 3D Printing by Two‐Photon Lithography
Source: Adv Sci (Weinh). 2025 Mar 31;12(21):2415159. doi: 10.1002/advs.202415159 (PMC12140314; doi:10.1002/advs.202415159)
Supplement: Supplementary file 1 — Supporting Information [file ADVS-12-2415159-s001.docx]

**Supporting Information**

**In Situ Control of Reactive Mesogens Alignment During 3D Printing by Two-Photon Lithography**

Tiziana Ritacco^1,2,3^, Alfredo Mazzulla^2^, Michele Giocondo^2^, Gabriella Cipparrone^1,2^ and Pasquale Pagliusi^1,2,^*.

*^1^Department of Physics, University of Calabria, 87036 Rende (CS), Italy*

*^2^CNR Nanotec – Institute of Nanotechnology, S.S. Cosenza, 87036 Rende (CS), Italy*

*^3^CNR IMM – Institute for Microelectronics and Microsystems S.S. Roma, 00133, Roma, Italy*

*Correspondent author:* [*pasquale.pagliusi@fis.unical.it*](mailto:pasquale.pagliusi@fis.unical.it)

S1. Spatial resolution of TPL-DiTuM microstructures in NRMs

The birefringent microstructures examined in this study (Figures 1-5) comprise single- or multi-layer arrays of parallel polymeric ribbons, varying in spacing and orientation relative to the anchoring axis (see **Figure S1**). These ribbons are fabricated by scanning a tightly focused near-infrared laser within a planar cell of nematic reactive mesogens (NRMs), manipulating the laser power (LP) and scan speed (SS). They represent the smallest feature achievable with two-photon photopolymerization lithography (TPL). In the following we present the morphological characterization of these ribbons, specifically their transverse dimensions (height and width), within the LP and SS ranges that enable the “director-tuning mode” (DiTuM).

Single-layer microdisks (diameter *d* = 50 μm), with a hatching spacing of H = 5.0 µm, are printed directly onto the aligning substrate, using laser power (LP = 7.5 – 12.5 mW) and scan speed (SS = 0.1 – 0.5 mm/s) parameters consistent with those used for the microdisks in Figures 2-4. The sample is then chemically developed by 25 minutes immersion in propylene glycol methyl ether acetate, to remove the unreacted NRMs and facilitate substrates separation. The substrate-supported TPL prints are then rinsed in an isopropanol bath for 5 minutes and air-dried at ambient temperature for 24 hours to evaporate residual solvent.

Atomic force microscopy (AFM; Bioscope Catalyst, Bruker-Nano) is used to measure the average height and width of the TPL-polymerized NRMs ribbons. The AFM system operated in non-contact mode, employing a silicon tip (radius 8 nm) on an antimony (n)-doped Si cantilever (model: RTESP-300, k = 40 N m^−1^, resonant frequency f_0_ = 300 kHz).

The ribbons’ dimensions are measured as depicted in **Figure S1**, which illustrates a 3D AFM image of a monolayer disk, and reported in **Table S1**.


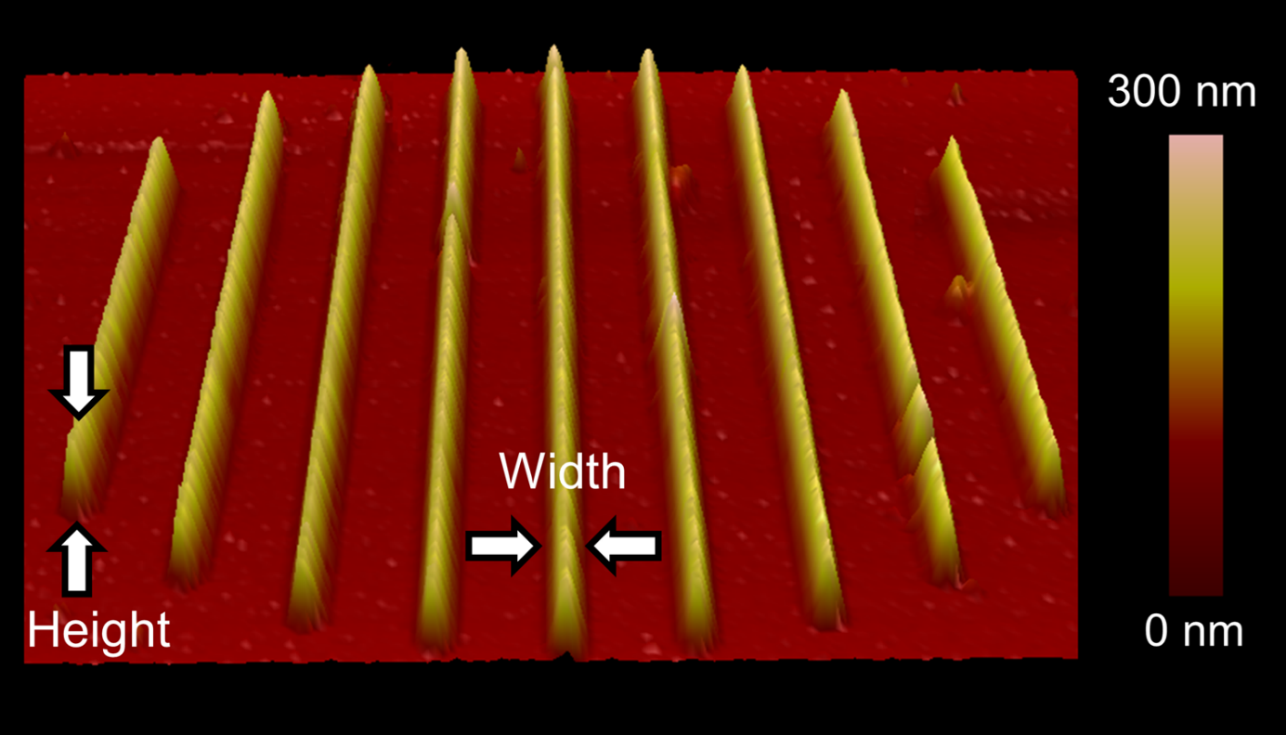


**Figure S1.** 3D AFM imaging of a microdisk (diameter *d* = 50 µm), hatched at 5.0 µm and printed in contact with the glass substrate.

|  | Height (nm) | | | | |  | | Width (nm) | | | | |  |
| --- | --- | --- | --- | --- | --- | --- | --- | --- | --- | --- | --- | --- | --- |
| SS (mm/s) | **0.1** | **0.2** | **0.3** | **0.4** | **0.5** | |  | **0.1** | **0.2** | **0.3** | **0.4** | **0.5** | |
| LP (mW) |  |  |  |  |  | |  |  |  |  |  |  | |
| 7.5 | 160 | 120 | 110 | 100 | 60 | |  | 250 | 240 | 210 | 190 | 180 | |
| 10.0 | 200 | 180 | 150 | 140 | 90 | |  | 290 | 240 | 240 | 200 | 180 | |
| 12.5 | 240 | 200 | 180 | 160 | 130 | |  | 340 | 280 | 240 | 230 | 190 | |

**Table S1.** Ribbons’ height and width are measured by AFM on chemically developed microdisks, with 5.0 µm spacing, for each combination of the LP and SS parameters. The average values are reported with a relative uncertainty of ~10%.

It is important to note that, to ensure the stability of the ribbons on the glass substrate, the laser beam is focused at the substrate surface. Therefore, the height of the substrate-attached ribbons corresponds to half the length of the minimum ellipsoidal volume, the “*voxel*”. Consequently, the heights of the ribbons printed above the surface, as in the experiments described in Sections 2.2-2.5 (see also Figures 3-5), are twice the height values reported in Table S1.

S2. Birefringence analysis of TPL-DiTuM microstructures in NRMs

We present an evaluation of the intrinsic birefringence *Δn* of single-layer microstructures fabricated in NRMs using TPL-DiTuM.

Previous work on multi-layer crisscrossed ribbon structures^[1]^ showed that laser power LP and scan speed SS do not affect the NRMs birefringence. In that case, however, the SS values (i.e. 4 – 10 mm/s) were sufficiently high to preserve local nematic director orientation. Here we aim to expand that study to include the low SS regime, specifically relevant to the DiTuM process.

Birefringence *Δn* is calculated starting from optical retardation *Γ* ≡ *h* *Δn* and the height *h* of the disks. Optical retardation *Γ* is measured on “optically developed” NRMs single-layer disks, printed at different hatching spacing H and direction ϑ (see Figure 1b). The measurements are performed using a Berek tilting compensator (5λ, Leitz Wetzlar) in cross-polarized optical microscopy, with the disks in the subtraction orientation (i.e., at α = 45° with respect to the polarizer and the analyzer), and with their slow axis perpendicular to the compensator’s one.^[2]^ The corresponding disks’ height *h* values are measured via AFM on chemically developed disks, as described in section S1.

The calculated birefringence *Δn* values show minimal variation across the explored range of TPL parameters, (**Figure S2**) and remain consistent with the nominal birefringence of a homogeneously aligned and UV-cured NRMs film (0.155±0.010), within experimental uncertainties.^[3]^ These findings demonstrate that, even in the DiTuM conditions, TPL does not induce appreciable changes in the intrinsic birefringence of the mesogenic material.


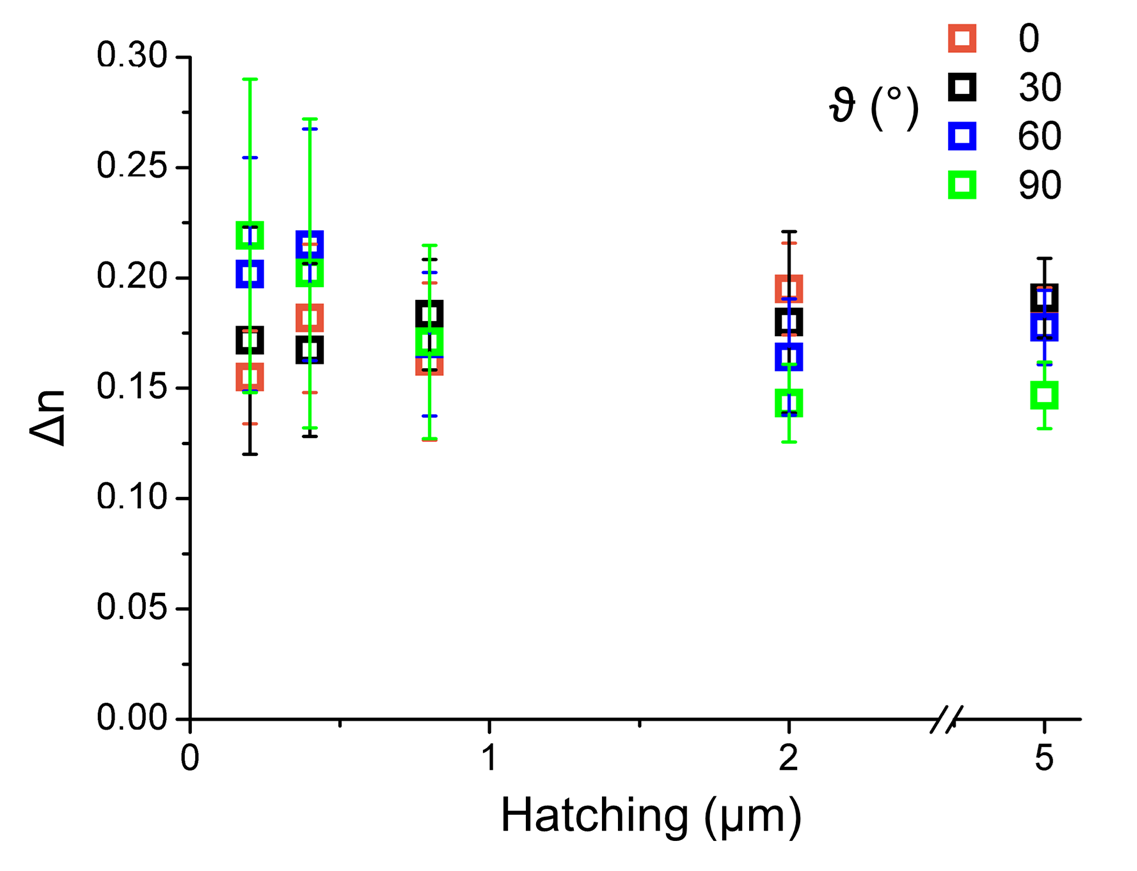


**Figure S2.** Birefringence of the single-layer disks plotted versus H, for different hatching directions ϑ.

S3. Form birefringence suppression by optical development.

Form birefringence occurs when light passes through structures characterized by a subwavelength repeating pattern, embedded in a medium with a different refractive index. The phenomenon arises from the differing boundary conditions imposed on electromagnetic waves propagating parallel and perpendicular to the structured material, even if the material itself is isotropic at the molecular scale. As a result, these structures exhibit a form birefringence that arises purely from their geometric arrangement.^[4-8]^ In our experiments, form birefringence would interfere with the intrinsic birefringence of the NRMs, related to molecular-scale anisotropy, and contribute to an “effective birefringence” of the structure as a whole. However, the present study is devoted to proving the capacity of TPL to reorient the average molecular alignment (director). To this end, we adopt an “optical development” method with the aim of suppressing the contribution of form birefringence in our grating-like microstructures.

In the following we verify that form birefringence can be effectively ruled out in “optically developed” subwavelength grating-like structures TPL-printed in an isotropic photoresist. Single-layer disks, varying in hatching spacing H and orientation ϑ (see Figures 1b), are fabricated in a commercial resist (IP-L 780, 95%, Nanoscribe GmbH), which exhibits no inherent birefringence. Before development, the disks are clearly visible in bright-field transmission microscopy (**Figure S3a**), due to the refractive index mismatch between the TPL-printed ribbons and the unpolymerized surrounding resist. Following UV curing of the latter, the disks are no longer discernible in both bright-field (Figure S3b) and cross-polarization (Figure S3c), confirming the absence of both refractive index inhomogeneity and form birefringence, respectively.


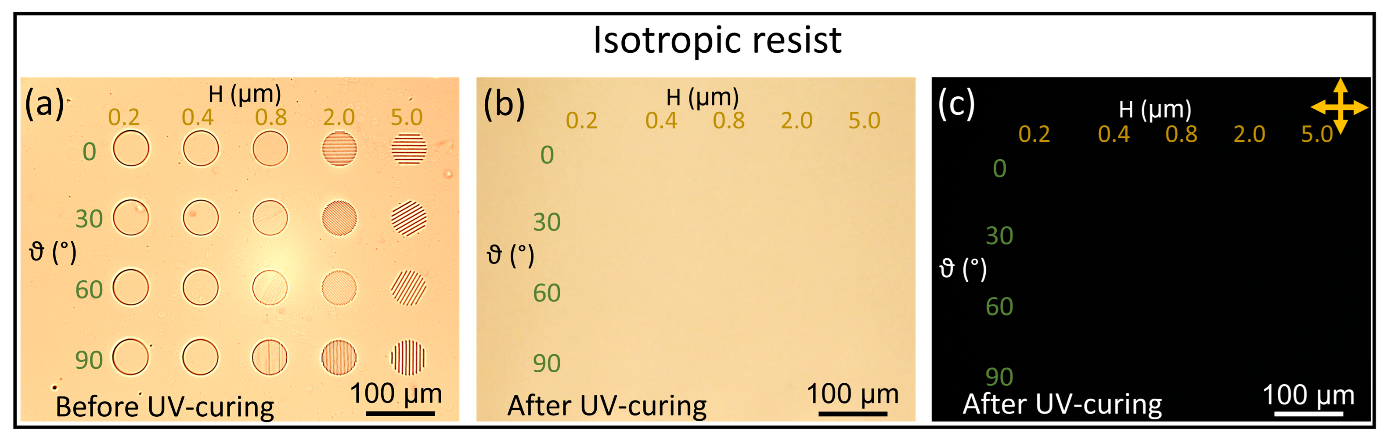


**Figure S3**. Transmission optical micrographs of the disks matrix TPL-printed in an isotropic resist at laser power 2.5 mW and scan speed 15 mm/s. Bright-field micrographs of the disks matrix are captured (a) before and (b) after UV curing the surrounding resist. (c) Micrograph of optically developed disks matrix between crossed polarizers confirms the absence of birefringence.

This evidence provides further support to the hypothesis that the optical anisotropy observed in polarization microscopy is exclusively due to the preferential orientation of the mesogenic moieties along the director, which defines the local optical axis.

**References**

[1] T. Ritacco, D. M. Aceti, G. De Domenico, M. Giocondo, A. Mazzulla, G. Cipparrone, P. Pagliusi. *Adv. Opt. Mater.* **2022**, *10, 2*, 2101526.

[2] G. Durey *Eur. Phys. J. Plus* **2021**, *136*, 866.

[3] Merck RMS03-001C datasheet, Merck KGaA (Darmstadt, Germany, **2006**).

[4] M. Born, E. Wolf. *Principles of Optics, 7th ed*., Section 15.5.2, Cambridge Univ. Press, **2006**.

[5] V. Twersky *J. Opt. Soc. Am.* **1975**, *65*, 239.

[6] C. Gu, P. Yeh. *Opt. Lett.* **1996**, *21*, 504.

[7] H. Kikuta, Y. Ohira, K. Iwata. *Appl. Opt.* **1997**, *36*, 1566.

[8] A. Emoto, M. Nishi, M. Okada, S. Manabe, S. Matsui, N. Kawatsuki, H. Ono. *Appl. Opt.* **2010**, *49, 23*, 4355.
